# *PLCE1* Polymorphisms and Risk of Esophageal and Gastric Cancer in a Northwestern Chinese Population

**DOI:** 10.1155/2019/9765191

**Published:** 2019-02-25

**Authors:** Ping Liang, Wentao Zhang, Weihua Wang, Peng Dai, Qin Wang, Wei Yan, Wei Wang, Xiaoying Lei, Daxiang Cui, Zhen Yan

**Affiliations:** ^1^State Key Laboratory of Cancer Biology, Department of Pharmacogenomics, School of Pharmacy, The Fourth Military Medical University, Xi'an 710032, China; ^2^Department of Pathology, Xijing Hospital, The Fourth Military Medical University, Xi'an 710032, China; ^3^Institute of Nano Biomedicine and Engineering, Key Laboratory for Thin Film and Microfabrication of Ministry of Education, Research Institute of Translation Medicine, Shanghai Jiao Tong University, Shanghai 200240, China

## Abstract

The reported risk susceptibility between* phospholipase C epsilon 1 (PLCE1) *polymorphisms and esophageal cancer (EC) and gastric cancer (GC) remained inconsistent and controversial, especially on variants other than rs2274223. The relationship between* PLCE1* polymorphisms and gene expression is also unclear. Here we conducted a case-control study from northwest China, genotyped seven tag single nucleotide polymorphisms (SNPs) in* PLCE1* with multiplexed SNP MassARRAY assay. Stratified analysis was carried out and* PLCE1* expression was evaluated in specified groups with the method of qRT-PCR and immunohistochemistry. Results showed that the minor alleles of rs3765524, rs2274223, and rs10509670 were associated with increased risk of EC and GC. Linkage disequilibrium analysis revealed protective haplotypes of CCAAGTC and CCAA. By stratification, a more significant association was found in subgroups of male, age ≥ 54, tumor stages of I-II and tumor size ≤ 5 cm, EC and cardia cancer (CC) of stomach, and moderate to well differentiated squamous carcinoma. In addition, a significant association for rs3765524 with noncardia cancer (NCC) and adenocarcinoma which is predominant in China was also observed. Further expression analysis identified that* PLCE1* was downregulated in NCC tissues comparing to their adjacent noncancerous tissues, and its protein expression was higher in genotype rs3765524 CT/TT than in rs3765524 CC. In summary, our study suggests that* PLCE1* polymorphisms may affect its gene expression and are associated with not only EC and CC, but also, to some extent, NCC risk in this study population.

## 1. Introduction

Esophageal cancer (EC) and gastric cancer (GC) are the two most common cancers originating from digestive tract around world [1], especially in China [2, 3]. There are many differences between EC and GC, such as genetic background, histological type, and* Helicobacter pylori* infection, while they are both known to be the results of complex interactions between inherited and environmental factors [4, 5].

*Phospholipase C epsilon 1 (PLCE1)* gene was reported to locate at 10q23, encoding a member of the human phosphoinositide-specific phospholipase C family [6]. It has been involved in the regulation of cell growth, differentiation, and oncogenesis [7]. Genome wide association studies (GWAS) have identified single nucleotide polymorphisms (SNPs), mostly rs2274223 A>G, and rs3765524 C>T in* PLCE1* gene as shared susceptibility loci for EC and GC [8–10].

Although several independent candidate-gene studies have confirmed the association between EC, GC, and* PLCE1* SNPs subsequently, there is more limited data on variants other than rs2274223, especially for GC [11–24]. Moreover, whether these loci are associated with noncardia cancer in addition to cardia and esophageal is not clear; whether or not* PLCE1* polymorphisms affect gene expression and protein function were only reported in few contradictory results [9, 14, 15, 23, 25–28].

To further explore the association between* PLCE1* polymorphisms and risk of EC and GC or their subtypes, we collected blood samples from Chinese northwestern population and used multiplexed SNP MassARRAY assay to sequence a panel of tag SNPs (tSNPs) of* PLCE1* in a case-control study. We completed a comprehensive analysis by logistic regression and stratification method and examined the expression of* PLCE1* in tissue samples.

## 2. Material and Methods

### 2.1. Study Population

A total of 324 GC or EC patients and 357 control volunteer individuals without known malignancies in the Xijing hospital of the Fourth Military Medical University in Xi'an city, China, during 2009 to 2012 were enrolled in the study. The cases had no previous history of other cancers, or prior chemotherapy or radiotherapy. All of the chosen subjects were Chinese Han living in Xi'an city and its surrounding areas. Generally, subjects with chronic diseases and conditions involving vital organs (heart, lung, liver, kidney, and brain) and severe endocrinological, metabolic, and nutritional diseases were excluded from this study. The purpose of the above exclusion procedures was to minimize the known environmental and therapeutic factors that influence the variation of human complex diseases. Peripheral blood samples from GC and EC patients were collected before or after surgery. Formalin-fixed, paraffin-embedded cancer and paired adjacent noncancerous tissues were collected after surgery from part of the GC patients. Patients' clinical data and postoperative pathological reports including the pathological types, pTNM and clinical stages, and the degrees of tumor differentiation were indexed from medical records. The study was approved by the Ethical Committee of Xijing Hospital (Xi'an, China), and this study complied with the World Medical Association Declaration of Helsinki. Informed consent was given by all the subjects for participation in this study.

### 2.2. DNA Isolation and Genotyping Assays

A panel of seven tSNPs of rs3765524, rs3818432, rs2274223, rs10509670, rs11187852, rs3781264, and rs11187866 in* PLCE1* gene were included in this study. All the SNPs have a disequilibrium (D′) threshold of 0.8 and minor allele frequency (MAF) > 0.05 in the HapMap Chinese Han population. Genomic DNA was extracted from peripheral blood using a Blood DNA Extraction Kit (TIANGEN, China), quantified with NanoDrop 2000 (Thermo, USA) and stored at −20°C until use. Primers were designed in a multiplexed SNP MassEXTEND assay with the Sequenom MassARRAY Assay Design 3.0 Software. SNP genotyping was performed by Sequenom MassARRAY RS1000 as reported previously [29]. Data management was conducted and analyzed by Sequenom Typer 4.0 Software.

### 2.3. Quantitative Real-Time PCR

Total RNA was extracted from tissue samples with E.Z.N.A.TM FFPE RNA Kit (OMEGA, USA). The protocol of total RNA isolation, cDNA preparation, and qRT-PCR was as reported previously by using the PrimeScriptTM RT Master Mix (Takara, Japan) on a 7500 fast real-time PCR system (Applied Biosystems) [29]. We used the following primers covering the two* PLCE1* spliceosomes, respectively:* PLCE1A*, forward 5′-ATCATAGAGACAGGCAGAGCACA-3′ and reverse 5′-ATGCCACATAGTTTTTCTTTTGC-3′;* PLCE1B*, forward 5′-GATTAATGGTTTCAGAAGGAAGTGC-3′ and reverse 5′- CTCCAGCATCCACATCCATCC-3′. Human* β-actin *was used as an endogenous control. For each sample, we calculated the difference in threshold cycles for each* PLCE1 *copy by the 2^−ΔCT^ method.

### 2.4. Immunohistochemistry Staining

The procedure of immunochemistry staining has been described in our previous publication [29]. Paraffin-embedded tissue specimens were deparaffinized in xylene and then soaked in ethanol and then PBS. We performed antigen retrieval in 100 mM sodium citrate buffer at 100°C for 20 min. Subsequently, we blocked endogenous peroxidase activity in 3% hydrogen peroxide in methanol for 15 min and then blocked nonspecific binding in 5% normal goat serum overnight at 4°C. We incubated sections for 2 hours at room temperature with rabbit anti-PLCE1 (SIGMA, HPA015598, 1:20 dilution) antibody, and then with alkaline phosphatase conjugated anti-rabbit IgG antibody. We visualized PLCE1 protein by Histostain™–Plus Kits (ZYMED, SP-9001). At least three experienced pathologists examined the staining using the following criteria: strong positive (signal in the cancer cells is stronger than the normal gastric gland), positive (signal in the cancer cells is as strong as that in a normal gastric gland), weak positive (signal between positive and negative), and negative (signal is no more than the background signal in the surrounding stromal cells).

### 2.5. Statistical Analysis

We performed statistical analysis using Microsoft Excel and SPSS 16.0 statistical package (SPSS, Chicago, IL). All *P* values in this study were two-sided. We considered* P* ≤ 0.05 the threshold for statistical significance. We tested genotypic frequencies in control subjects for each SNP for departure from HWE using an exact test. We compared genotype frequencies of case and control subjects using the Chi^2^ test. We calculated OR and 95% CI by unconditional logistic regression analysis. There were two factors of age and gender adjusted for the analysis. We used the Haploview program to estimate the pairwise LD between markers and partition haplotype blocks. We inferred haplotypes using the Haploview software package (version 4.2).

## 3. Results

### 3.1. Overall Association between the PLCE1 tSNPs and the Risk of EC and GC

The characteristics of all cases and controls included in the study were listed in Table 1. Seven tSNPs in* PLCE1* gene were genotyped and all of the tSNPs were in Hardy-Weinberg equilibrium (HWE) in the control population (*P* > 0.05).

After genotyping, we conducted logistic regression to evaluate the association of each tSNP with the risk of EC and GC (Table 2). Results showed that there were three tSNPs (rs3765524, rs2274223, and rs10509670) associated with the risk of EC and GC: rs3765524 (CT vs CC, OR = 1.66, 95% CI 1.16-2.38,* P* = 0.006; CT/TT vs CC, OR = 1.65, 95% CI 1.17-2.34,* P* = 0.004); rs2274223 (AG vs AA, OR = 1.57, 95% CI 1.10-2.26,* P* = 0.014; AG/GG vs AA, OR = 1.55, 95% CI 1.10-2.20,* P* = 0.013); rs10509670 (AG vs AA, OR = 1.54, 95% CI 1.07-2.21,* P* = 0.019; AG/GG vs AA, OR = 1.54, 95% CI 1.09-2.18,* P* = 0.014).

### 3.2. Linkage Disequilibrium and Haplotype Evaluation for the PLCE1 tSNPs

**Figure 1:**
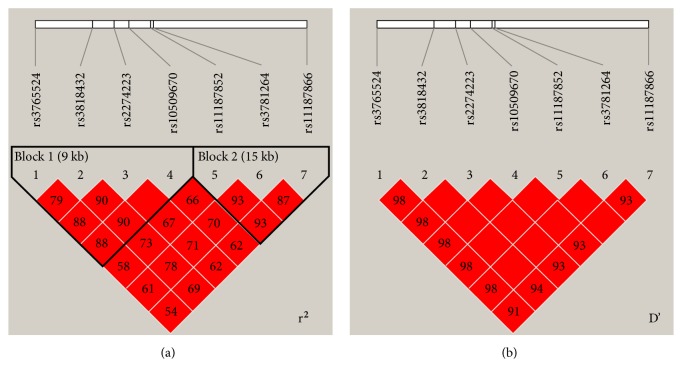
*Linkage disequilibrium (LD) analysis of PLCE1 tSNPs*. (a) r^2^ of LD analysis, which showed that the seven tSNPs are linked to each other and could be divided into two subblocks. (b) D' of LD analysis.

Linkage disequilibrium (LD) analysis revealed that the seven tSNPs of* PLCE1 *linked with each other (Figure 1). Haplotype “CCAAGTC” accounted for 71.5% of the whole haplotypes in EC and GC cases. This is a protective haplotype against the risk of EC/GC (OR = 0.72; 95% CI = 0.53–0.97;* P* = 0.029) (Table 3). Further analysis revealed that the LD block could be divided into two subblocks (Figure 1(a)). Subblock 1 (r^2^ > 0.79) was composed of four tSNPs of rs3765524, rs3818432, rs2274223, and rs10509670, where the three risk SNPs identified above were included. Subblock 2 (r^2^ > 0.87) included the later three tSNPs of rs11187852, rs3781264, and rs11187866. In subblock 1, “CCAA” accounted for 72.4% of the whole haplotypes in EC/GC cases and was found to be the protective haplotype against the risk of EC/GC (OR = 0.67; 95% CI = 0.49–0.91;* P* = 0.009).

### 3.3. Stratified Analysis for the Clinicopathologic Data of Patients

Anatomically, gastric cancer includes cardia cancer (CC) and noncardia cancers (NCC). Pathologically, gastric cancer has adenocarcinoma and squamous carcinoma. Then we performed a stratified analysis to determine the association between the three tSNPs (rs3765524, rs2274223, and rs10509670) and clinicopathologic data in dominant model (Table 4). Significant association between the three tSNPs and risk of EC and GC was observed for subgroup patients of male, age ≥54, tumor stages of I-II and tumor size ≤ 5 cm, EC and cardia cancer (CC), and moderate to well differentiated squamous carcinoma. In addition, a significant association for rs3765524 with noncardia cancer (NCC) and adenocarcinoma was also observed.

### 3.4. Expression Distribution of PLCE1 Protein in Stomach Tissue

**Figure 2:**
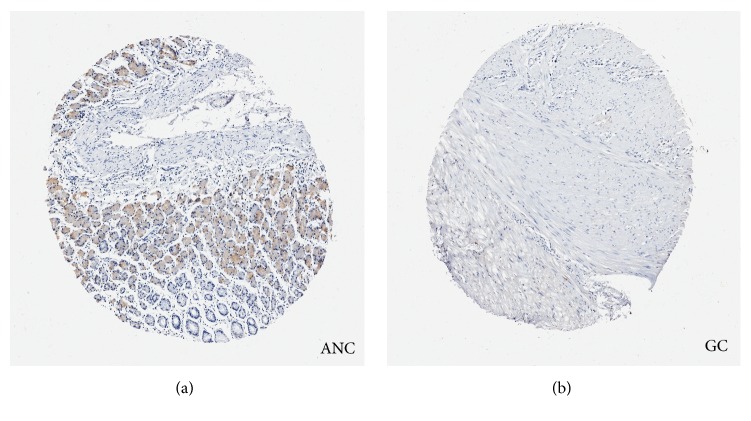
*Representative images of PLCE1 expression distribution in gastric cancer (GC) and adjacent noncancerous (ANC) tissues by tissue microarray*. (a) ANC tissues. PLCE1 protein was expressed in the cytoplasm of columnar epithelial cells and mainly distributed in the junction of cardia and gastric fundus glands (200×). (b) GC tissues. Structure distortion and confusion of tubular glands, heterogeneity of epithelial cells with irregular nuclear staining, and lower expression of PLCE1 were observed (200×).

Now that the association between* PLCE1* polymorphisms and GC risk exhibited disparity according to the tumor subsites, we then evaluated the expression distribution of PLCE1 protein in human GC and adjacent noncancer tissues (ANC) by tissue microarray. In the ANC tissue, PLCE1 protein expression was positive in the cytoplasm of columnar epithelial cells and mainly distributed in the junction of cardia and gastric fundus glands (Figure 2(a)). In the GC tissue, the structure distortion and confusion of tubular glands, obvious heterogeneity of epithelial cells with irregular nuclear staining, and lower expression of PLCE1 protein were observed (Figure 2(b)). These suggested, together with the results of rs3765524 genotyping by stratified analysis, that PLCE1 protein may be involved in carcinogenesis of NCC and adenocarcinoma, although more significant association has been found with EC, CC, and squamous carcinoma.

### 3.5. Effect of PLCE1 Polymorphisms on Its Expression in NCC and ANC Tissues

**Figure 3:**
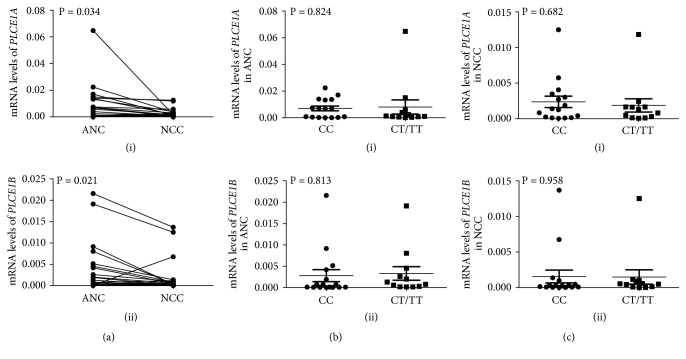
*PLCE1 mRNA expression in adenocarcinoma of noncardia cancer (NCC) and adjacent noncancerous (ANC) tissues by qRT-PCR*. (a)* PLCE1A* (i) and* PLCE1B* (ii) mRNA expression in ANC and NCC tissues, respectively. (b)* PLCE1A* (i) and* PLCE1B* (ii) mRNA expression in ANC tissues with different genotypes of rs3765524. (c)* PLCE1A* (i) and* PLCE1B* (ii) mRNA expression in NCC tissues with different genotypes of rs3765524. Horizontal lines indicate mean ± SE (n = 28).

Because NCC and adenocarcinoma have been the predominant subtype of gastric cancer in China, we then examined the expression of* PLCE1* in adenocarcinoma of NCC and their ANC tissues with different genotypes of rs3765524. For mRNA transcription, we identified two* PLCE1* processing units,* PLCE1A* and* PLCE1B*, by quantitative real-time PCR (qRT-PCR). As presented in Figures 3(a)(i) and 3(a)(ii), compared with ANC tissues, the two procession units were both downregulated in NCC tissues (*PLCE1A*: 22 of 28, 78.57%,* P* = 0.034;* PLCE1B*: 21 of 28, 75.00%,* P* = 0.021), while there was no significant difference between genotype rs3765524 CC and rs3765524 CT/TT both in ANC (Figures 3(b)(i) and 3(b)(ii)) and in NCC (Figures 3(c)(i) and 3(c)(ii)).

**Figure 4:**
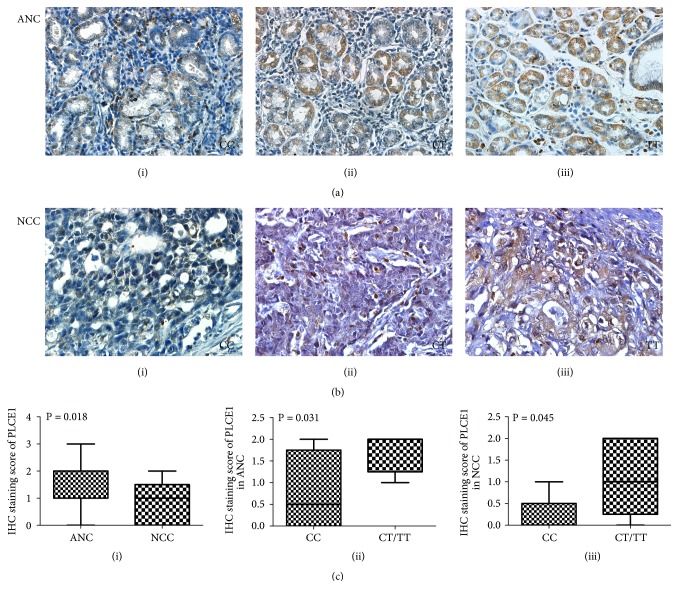
*PLCE1 protein expression in adenocarcinoma of noncardia cancer (NCC) and adjacent noncancerous (ANC) tissues by immunohistochemistry (IHC) staining*. (a) Representative images of PLCE1 expression in ANC tissues with different genotypes of rs3765524 ((i) CC, (ii) CT, and (iii) TT) (400×). (b) Representative images of PLCE1 expression in NCC tissues with different genotypes of rs3765524 ((i) CC, (ii) CT, and (iii) TT) (400×). (c) IHC staining score comparison of PLCE1 (i) between ANC and NCC tissues regardless of rs3765524 genotyping, (ii) in ANC tissues with different genotypes of rs3765524, (iii) in NCC tissues with different genotypes of rs3765524. The data was represented as mean ± SE (n = 13).

For protein translation, immunohistochemistry (IHC) staining revealed that PLCE1 protein expression was generally downregulated in NCC than in their ANC tissues regardless of rs3765524 genotype (6 of 13, 46.15%,* P* = 0.018, see in Figures 4(a), 4(b), and 4(c)(i)). By genotyping, the PLCE1 protein expression was found higher in group of rs3765524 CT/TT than in group of rs3765524 CC both in ANC (Figure 4(c)(ii).* P* = 0.031) and NCC tissues (Figure 4(c)(iii).* P* = 0.045).

## 4. Discussion

SNPs are the most common type of genetic variation, which makes them excellent biological markers [30]. On the other hand, SNPs, including those that fall within the coding or noncoding regions of genes, may affect the gene transcription and translation, as well as the structure and function of protein, contributing to changing the host susceptibility to diseases [31].

GWAS study found that some SNPs in* PLCE1* corresponding to Y, C2, and RA domain were associated with the risk of EC and GC [8–10]. These are very important domains to PLCE1. The Y domain folds to form the catalytic core of the phospholipase and the C2 domain can bind to phospholipid [32]. RA domain is in the C terminal of PLCE1 protein, which interacts directly with upstream regulators of Ras, Rap, and others [33]. The genomic region for Y, C2, and RA domains spans from exon 24 to exon 33. By referring to the frequencies of SNPs in Chinese Han population in HapMap database, after removing the SNPs with minimum allele frequency (MAF) less than 0.05, seven candidate SNPs in the region were selected in our study, where rs3765524 was in exon 24 and in Y domain, rs3818432 was in intron 24, rs2274223 was in exon 26 and in C2 domain, rs10509670 was in intron 26, rs11187852 and rs3781264 were in intron 27, and rs11187866 was in intron 32.

By genotyping and logistic regression, we not only confirmed the two previous reported SNPs of rs3765524 and rs2274223 [8–10] but also revealed that another SNP of rs10509670 in* PLCE1* was associated with the risk of EC and GC susceptibility. rs3765524 C>T causes an amino acid change from Thr to Ile (ACC1777ATC), and rs2274223 A>G can also cause a missense mutation of His to Arg (CAC1927CGC). These two SNPs are corresponding to the Y and C2 domain of PLCE1 protein, respectively. We noticed that Thr, His, and Arg are frequently modified amino acid residues in human proteins. Different posttranslational modification may alter the structure, stability, and function of PLCE1 protein [34]. In the case of rs3765524, we found that although there was no difference in mRNA transcription between wild type and mutant type (Figure 3), there was a difference in protein expression (Figure 4). Among them, the expression of CT/TT genotype was higher than that of CC genotype in both NCC and ANC groups, implying that the amino acid change by the polymorphism of rs3765524 might lead to different protein modifications or structural changes, ultimately affecting* PLCE1* expression or stability.

The third loci of rs10509670 located in the intron of* PLCE1* gene has also shown to be associated with risk of EC and GC in the experiment. We hypothesize that rs10509670 A>G may affect* PLCE1* gene structure or expression by regulating gene splicing or transcription [31]. In the study, the seven tSNPs have been proved to be in LD. Moreover, we identified two haplotypes associated with EC and GC risk. The haplotype of “CCAAGTC” (corresponding to Y, C2, and RA domains) and the haplotype in subblock 1 of “CCAA” (corresponding to Y and C2 domains) have decreased risk of EC and GC of 33% and 28%, respectively.

Previous studies have exhibited different associations between* PLCE1* polymorphisms and the risk of EC and GC, especially for different tumor subsites of GC in several candidate-gene studies [11–24]. The latest large meta-analyses confirmed the G allele of* PLCE1 *rs2274223 to be associated with an increased risk of cardia cancer (CC) rather than noncardia cancer (NCC) [35]. In our stratification analysis, we not only confirmed the T allele of rs3765524 and G allele of rs2274223 but also identified that the G allele of rs10509670 was associated with increased risk of EC and CC susceptibility. Furthermore, we revealed a significant association of rs3765524 C>T with the increased risk of NCC and adenocarcinoma. As we know, NCC has predominant incidence among digestive tract tumors in China [36, 37].

So far, the literature reports about* PLCE1* expression and distribution were still unclear and conflicting. Previously, we conducted a comprehensive analysis of* PLCE1 *expression in atrophic gastritis and GC tissues, which revealed that differential expression of* PLCE1* may distinguish GC from inflammation lesions [28]. In terms of tumorous-normal comparison, upregulation and downregulation of* PLCE1* were both found in EC and CC at mRNA and/or protein levels [9, 15, 25, 26], while there was only one study that identified downregulation of* PLCE1* at mRNA level in NCC [26]. In terms of the comparison of minor-major alleles of rs2274223 with* PLCE1* expression, the results for EC were also inconsistent [14, 15, 38], and there is no report about CC and NCC until now. Another two studies reported the expression of* PLCE1* in GC but without specific tumor subsites information (CC or NCC), which presented opposite conclusions for tumorous to normal comparison [23, 28].

By tissue microarray, we identified that PLCE1 protein is expressed not only in cardia but also in gastric fundus glands both in GC and ANC tissues. This result, together with the association of rs3765524 C>T with NCC risk, suggests that PLCE1 protein may be involved in carcinogenesis of NCC. Therefore, we used qRT-PCR and IHC to study genetic variation effects on* PLCE1* expression in NCC and their ANC tissues. Results showed that the expression of* PLCE1* at both mRNA and protein levels was lower in NCC tissues than in their ANC tissues, which supports the hypothesis that PLCE1 may function as a tumor suppressor. We also found that rs3765524 genotype may affect* PLCE1* expression, where* PLCE1* expression was higher in group of rs3765524 CT/TT than in group of CC. This strongly suggests, as one of the contributors, the reference allele C of rs3765524 loss of expression in tumor, but the mutated T allele, on the other hand, produces a “dominant negative” phenotype, which is related to the increased risk of NCC. Of course, the exact mechanism needs to be studied further. To our knowledge, this is the first report about* PLCE1* expression distribution in NCC by genotypes.

PLCE1A and PLCE1B arise from alternative splicing at the amino terminus of PLCE1 protein. PLCE1A is composed of 2303aa. PLCE1B is composed of 1994aa which is truncated at the amino terminal of the peptide [39]. The different distribution and function of the two subunits in gastric carcinogenesis have not been studied yet. We demonstrated, through qRT-PCR, that both* PLCE1A *and* PLCE1B* were downregulated in NCC than their ANC tissues. This suggests that* PLCE1A *and* PLCE1B *may be involved in NCC carcinogenesis.

## 5. Conclusion

Our study reveals that* PLCE1* polymorphisms may affect gene expression and function and are associated with the risk of not only EC and CC, but also, to some extent, NCC in northwestern Chinese population. The tSNPs of* PLCE1* may have a potential possibility to be biomarkers for prewarning and diagnosis against these diseases.

## Figures and Tables

**Table 1:** Characteristics of case and control.

Variables	Case (324)		Control (357)	
	No. (%)		No. (%)	
Age, mean years (SD)	57.4	(11.4)	50.4	(12.7)
Sex				
Male	252	(77.8)	212	(59.4)
Female	72	(22.2)	145	(40.6)
Tumor site				
EC	48	(14.8)		
CC	35	(10.8)		
NCC	241	(74.4)		
Pathology				
Adenocarcinoma	223	(68.8)		
Squamous carcinoma	38	(11.7)		
Unspecified	63	(19.4)		
Differentiation				
Poor	117	(36.1)		
Moderate to well	117	(36.1)		
Unspecified	90	(27.8)		
Tumor size				
≤5cm	153	(36.2)		
>5cm	71	(16.8)		
Unspecified	199	(47.0)		
Tumor stage				
I-II	174	(53.7)		
III-IV	62	(19.1)		
Unspecified	88	(27.2)		

EC, esophageal cancer; CC, cardia cancer; NCC, noncardia cancer.

**Table 2:** Logistic regression of candidate tSNPs in *PLCE1* and their association with EC and GC risk.

ID	Genotype	Case (n = 324)		Control (n = 357)		Crude OR		*P*	Adjusted OR		*P*	HWE-*P *
		No. (%)		No. (%)		(95%CI)			(95%CI)			
rs3765524	CC	168	(51.9)	223	(62.6)	1			1			0.896
	CT	136	(42.0)	117	(32.9)	1.54	(1.12-2.12)	**0.008 **	1.66	(1.16-2.38)	**0.006 **	
	TT	20	(6.2)	16	(4.5)	1.66	(0.83-3.30)	0.149	1.60	(0.72-3.55)	0.247	
	CT/TT	156	(48.1)	133	(37.4)	1.56	(1.15-2.11)	**0.005 **	1.65	(1.17-2.34)	**0.004 **	
rs3818432	CC	104	(57.5)	193	(65.6)	1			1			0.856
	CA	73	(40.3)	91	(31.0)	1.49	(1.01-2.20)	**0.045 **	1.47	(0.97-2.23)	0.073	
	AA	4	(2.2)	10	(3.4)	0.74	(0.23-2.42)	0.622	0.87	(0.24-3.09)	0.826	
	CA/AA	77	(42.5)	101	(34.4)	1.41	(0.97-2.07)	0.074	1.41	(0.94-2.13)	0.098	
rs2274223	AA	177	(54.8)	229	(64.1)	1			1			0.457
	AG	126	(39.0)	111	(31.1)	1.47	(1.06-2.03)	**0.019 **	1.57	(1.10-2.26)	**0.014 **	
	GG	20	(6.2)	17	(4.8)	1.52	(0.77-2.99)	0.223	1.43	(0.66-3.14)	0.367	
	AG/GG	146	(45.2)	128	(35.9)	1.48	(1.08-2.01)	**0.013 **	1.55	(1.10-2.20)	**0.013 **	
rs10509670	AA	178	(54.9)	229	(64.5)	1			1			0.391
	AG	124	(38.3)	109	(30.7)	1.46	(1.06-2.02)	**0.021 **	1.54	(1.07-2.21)	**0.019 **	
	GG	22	(6.8)	17	(4.8)	1.66	(0.86-3.23)	0.132	1.54	(0.72-3.32)	0.268	
	AG/GG	146	(45.1)	126	(35.5)	1.49	(1.10-2.03)	**0.011 **	1.54	(1.09-2.18)	**0.014 **	
rs11187852	GG	122	(67.4)	212	(72.1)	1			1			0.506
	GA	56	(30.9)	77	(26.2)	1.26	(0.84-1.90)	0.263	1.33	(0.85-2.07)	0.212	
	AA	3	(1.7)	5	(1.7)	1.04	(0.24-4.44)	0.955	1.68	(0.34-8.18)	0.522	
	GA/AA	59	(32.6)	82	(27.9)	1.25	(0.84-1.87)	0.276	1.34	(0.87-2.08)	0.185	
rs3781264	TT	120	(66.3)	206	(70.1)	1			1			0.511
	TC	58	(32.0)	82	(27.9)	1.21	(0.81-1.82)	0.347	1.24	(0.80-1.92)	0.331	
	CC	3	(1.7)	6	(2.0)	0.86	(0.21-3.49)	0.831	1.07	(0.23-5.00)	0.932	
	TC/CC	61	(33.7)	88	(29.9)	1.19	(0.80-1.77)	0.390	1.23	(0.80-1.89)	0.340	
rs11187866	CC	120	(66.7)	210	(71.4)	1			1			0.723
	CG	56	(31.1)	76	(25.9)	1.29	(0.85-1.95)	0.226	1.33	(0.85-2.08)	0.210	
	GG	4	(2.2)	8	(2.7)	0.88	(0.26-2.97)	0.830	1.13	(0.30-4.23)	0.857	
	CG/GG	60	(33.3)	84	(28.6)	1.25	(0.84-1.86)	0.274	1.31	(0.85-2.03)	0.218	

*Notes.* The adjusted OR is derived from the correction for age and gender. The results were in bold, if the *P* value < 0.05.

EC, esophageal cancer; GC, gastric cancer.

**Table 3:** Haplotypes of *PLCE1* and their association with EC and GC risk.

ID	Block	Haplotype	Frequency^a^		OR	(95%CI)	Chi^2^	*P* ^b^
			Case	Control				
1	Global	CCAAGTC	71.5%	77.8%	0.72	(0.53-0.97)	4.742	**0.029 **
2		TAGGACG	16.8%	14.6%	1.18	(0.83-1.69)	0.861	0.354
3		TAGGGTC	4.7%	2.9%	1.66	(0.83-3.28)	2.115	0.146
4		TCAAGTC	3.0%	1.2%	2.49	(0.97-6.41)	3.834	0.050
5		TCGGGTC	2.2%	1.2%	1.82	(0.66-5.03)	1.381	0.240
6	Sub-1	CCAA	72.4%	79.7%	0.67	(0.49-0.91)	6.756	**0.009 **
7		TAGG	22.1%	17.6%	1.33	(0.96-1.84)	2.859	0.091
8		TCAA	3.0%	1.2%	2.48	(0.95-6.42)	3.752	0.053
9		TCGG	2.2%	1.2%	1.81	(0.65-5.04)	1.331	0.249
10	Sub-2	GTC	81.5%	83.2%	0.89	(0.63-1.25)	0.436	0.509
11		ACG	17.1%	14.8%	1.19	(0.83-1.70)	0.921	0.337

*Notes.* (A) Only haplotypes with frequencies of ≥ 3% are shown. (B) The results were in bold, if the *P* value < 0.05.

EC, esophageal cancer; GC, gastric cancer.

**Table 4:** Stratified analysis for the association between tSNPs in *PLCE1* and EC and GC risk.

Variables	rs3765524		Adjusted OR		*P*	rs2274223		Adjusted OR		*P*	rs10509670		Adjusted OR		*P*
	(Case/Control)					(Case/Control)					(Case/Control)				
	CC	CT/TT	(95%CI)			AA	AG/GG	(95%CI)			AA	AG/GG	(95%CI)		
Age, years															
≤ 53	58/159	55/109	1.34	(0.86-2.10)	0.203	62/161	50/107	1.18	(0.75-1.86)	0.464	63/161	50/105	1.18	(0.75-1.85)	0.478
>53	110/64	101/24	2.56	(1.41-4.64)	**0.002 **	115/68	96/21	2.63	(1.43-4.82)	**0.002 **	115/68	96/21	2.63	(1.43-4.82)	**0.002 **
Sex															
Male	130/137	122/74	1.84	(1.19-2.82)	**0.006 **	136/139	116/73	1.72	(1.12-2.64)	**0.014 **	135/139	117/72	1.76	(1.15-2.71)	**0.010 **
Female	38/86	34/59	1.34	(0.74-2.42)	0.339	41/90	30/55	1.25	(0.68-2.28)	0.476	43/90	29/54	1.16	(0.63-2.12)	0.636
Tumor site															
EC	25/223	23/133	2.61	(1.22-5.55)	**0.013 **	24/229	23/128	3.01	(1.39-6.50)	**0.005 **	25/229	23/126	1.40	(1.05-1.86)	**0.022 **
CC	11/223	24/133	5.99	(2.28-15.77)	**<0.001**	12/229	23/128	5.31	(2.09-13.50)	**<0.001**	12/229	23/126	5.31	(2.09-13.50)	**<0.001**
NCC	142/223	114/133	1.47	(1.03-2.12)	**0.036 **	152/229	104/128	1.36	(0.95-1.96)	0.098	152/229	104/126	1.36	(0.95-1.96)	0.096
Pathology															
Adenocarcinoma	172/223	141/133	1.45	(1.02-2.06)	**0.041 **	181/229	132/128	1.36	(0.95-1.94)	0.092	180/229	133/126	1.38	(0.97-1.97)	0.078
Squamous carcinoma	21/223	20/133	2.23	(1.05-4.72)	**0.037 **	20/229	21/128	2.71	(1.28-5.76)	**0.010 **	20/229	21/126	2.72	(1.28-5.77)	**0.009 **
Differentiation															
Poor	85/223	59/133	1.30	(0.85-1.97)	0.222	86/229	58/128	1.33	(0.87-2.03)	0.182	86/229	58/126	1.40	(1.05-1.86)	**0.022 **
Moderate to well	92/223	83/133	1.72	(1.07-2.76)	**0.026 **	97/229	78/128	1.61	(1.00-2.58)	**0.050 **	96/229	79/126	1.67	(1.04-2.68)	**0.034 **
Tumor size															
≤ 5 cm	104/223	100/133	1.81	(1.20-2.72)	**0.005 **	114/229	90/128	1.59	(1.05-2.39)	**0.027 **	115/229	89/126	1.54	(1.02-2.32)	**0.039 **
> 5 cm	58/223	43/133	1.44	(0.88-2.34)	0.148	60/229	41/128	1.39	(0.85-2.27)	0.192	60/229	41/126	1.40	(0.85-2.28)	0.183
Tumor stage															
I-II	129/223	115/133	1.60	(1.09-2.33)	**0.016 **	136/229	108/128	1.50	(1.03-2.19)	**0.036 **	137/229	107/126	1.47	(1.01-2.15)	**0.047 **
III-IV	45/223	32/133	1.38	(0.79-2.40)	0.262	47/229	30/128	1.35	(0.77-2.36)	0.297	45/229	32/126	1.53	(0.88-2.68)	0.134

*Notes.* The adjusted OR is derived from the correction for age and gender. The results were in bold, if the *P* value < 0.05.

EC, esophageal cancer; GC, gastric cancer; CC, cardia cancer; NCC, noncardia cancer.

## Data Availability

The data used to support the findings of this study are available from the corresponding author upon request.

## References

[1] Bray F., Ferlay J., Soerjomataram I. (2018). Global cancer statistics 2018: GLOBOCAN estimates of incidence and mortality worldwide for 36 cancers in 185 countries. *CA: A Cancer Journal for Clinicians*.

[2] Chen W., Zheng R., Baade P. D. (2016). Cancer statistics in China, 2015. *CA: A Cancer Journal for Clinicians*.

[3] Cui D.-X., Zhang L., Yan X.-J. (2005). A microarray-based gastric carcinoma prewarning system. *World Journal of Gastroenterology*.

[4] Cheung W. Y., Liu G. (2009). Genetic variations in esophageal cancer risk and prognosis. *Gastroenterology Clinics of North America*.

[5] Hartgrink H. H., Jansen E. P., van Grieken N. C., van de Velde C. J. (2009). Gastric cancer. *The Lancet*.

[6] Song C., Hu C. D., Masago M. (2001). Regulation of a novel human phospholipase C, PLCepsilon, through membrane targeting by Ras. *The Journal of Biological Chemistry*.

[7] Citro S., Malik S., Oestreich E. A. (2007). Phospholipase Cepsilon is a nexus for Rho and Rap-mediated G protein-coupled receptor-induced astrocyte proliferation. *Proceedings of the National Acadamy of Sciences of the United States of America*.

[8] Abnet C. C., Freedman N. D., Hu N. (2010). A shared susceptibility locus in PLCE1 at 10q23 for gastric adenocarcinoma and esophageal squamous cell carcinoma. *Nature Genetics*.

[9] Wang L. D., Zhou F. Y., Li X. M. (2010). Genome-wide association study of esophageal squamous cell carcinoma in Chinese subjects identifies susceptibility loci at PLCE1 and C20orf54. *Nature Genetics*.

[10] Wu C., Hu Z., He Z. (2011). Genome-wide association study identifies three new susceptibility loci for esophageal squamous-cell carcinoma in Chinese populations. *Nature Genetics*.

[11] Jia X., Liu P., Zhang M. (2015). Genetic variants at 6p21, 10q23, 16q21 and 22q12 are associated with esophageal cancer risk in a Chinese Han population. *International Journal of Clinical and Experimental Medicine*.

[12] Piao J.-M., Shin M.-H., Kim H. N. (2014). Replication of results of genome-wide association studies on esophageal squamous cell carcinoma susceptibility loci in a Korean population. *Diseases of the Esophagus*.

[13] Duan F., Xie W., Cui L. (2013). Novel functional variants locus in PLCE1 and susceptibility to esophageal squamous cell carcinoma: Based on published genome-wide association studies in a central Chinese population. *Cancer Epidemiology*.

[14] Cui X.-B., Chen Y.-Z., Pang X.-L. (2013). Multiple polymorphisms within the PLCE1 are associated with esophageal cancer via promoting the gene expression in a Chinese Kazakh population. *Gene*.

[15] Hu H., Yang J., Sun Y. (2012). Putatively functional PLCE1 variants and susceptibility to esophageal squamous cell carcinoma (ESCC): a case-control study in eastern Chinese populations. *Annals of Surgical Oncology*.

[16] Zhou R., Li Y., Wang N., Liu B., Chen Z., Zuo L. (2012). PLC-*ε*1 gene polymorphisms significantly enhance the risk of esophageal squamous cell carcinoma in individuals with a family history of upper gastrointestinal cancers. *Archives of Medical Research*.

[17] Gu H., Ding G., Zhang W. (2012). Replication study of PLCE1 and C20orf54 polymorphism and risk of esophageal cancer in a Chinese population. *Molecular Biology Reports*.

[18] Yuan J., Li Y., Tian T. (2016). Risk prediction for early-onset gastric carcinoma: a case-control study of polygenic gastric cancer in Han Chinese with hereditary background. *Oncotarget *.

[19] Sun H., Wu X., Wu F. (2015). Associations of genetic variants in the PSCA, MUC1 and PLCE1 genes with stomach cancer susceptibility in a Chinese population. *PLoS ONE*.

[20] Mou X., Li T., Wang J. (2015). Genetic variation of BCL2 (rs2279115), NEIL2 (rs804270), LTA (rs909253), PSCA (rs2294008) and PLCE1 (rs3765524, rs10509670) genes and their correlation to gastric cancer risk based on universal tagged arrays and Fe3O4 magnetic nanoparticles. *Journal of Biomedical Nanotechnology*.

[21] He Y., Wang C., Wang Z., Zhou Z. (2016). Genetic variant PLCE1 rs2274223 and gastric cancer: more to be explored?. *Gut*.

[22] Li M., Huang L., Qiu H. (2013). Helicobacter pylori infection synergizes with three inflammation-related genetic variants in the GWASs to increase risk of gastric cancer in a Chinese population. *PLoS ONE*.

[23] Wang M., Zhang R., He J. (2012). Potentially functional variants of PLCE1 identified by GWASs contribute to gastric adenocarcinoma susceptibility in an Eastern Chinese population. *PLoS ONE*.

[24] Zhang H., Jin G., Li H. (2011). Genetic variants at 1q22 and 10q23 reproducibly associated with gastric cancer susceptibility in a Chinese population. *Carcinogenesis*.

[25] Cui X.-B., Pang X.-L., Li S. (2014). Elevated expression patterns and tight correlation of the PLCE1 and NF-*κ*B signaling in Kazakh patients with esophageal carcinoma. *Medical Oncology*.

[26] Li W., Hu N., Burton V. H. (2014). PLCE1 mRNA and protein expression and survival of patients with esophageal squamous cell carcinoma and gastric adenocarcinoma. *Cancer Epidemiology Biomarkers & Prevention*.

[27] Chen Y.-Z., Cui X.-B., Hu J.-M. (2013). Overexpression of PLCE1 in Kazakh esophageal squamous cell carcinoma: Implications in cancer metastasis and aggressiveness. *APMIS-Acta Pathologica, Microbiologica et Immunologica Scandinavica*.

[28] Chen J., Wang W., Zhang T. (2012). Differential expression of phospholipase C epsilon 1 is associated with chronic atrophic gastritis and gastric cancer. *PLoS ONE*.

[29] Zhang W., Liang P., Wang W. (2015). The influence of PSCA gene variation on its expression and gastric adenocarcinoma susceptibility in the northwest chinese population. *International Journal of Molecular Sciences*.

[30] Engle L. J., Simpson C. L., Landers J. E. (2006). Using high-throughput SNP technologies to study cancer. *Oncogene*.

[31] Salisbury B. A., Pungliya M., Choi J. Y., Jiang R., Sun X. J., Stephens J. (2003). SNP and haplotype variation in the human genome. *Mutation Research - Fundamental and Molecular Mechanisms of Mutagenesis*.

[32] Harden T. K., Sondek J. (2006). Regulation of phospholipase C isozymes by ras superfamily GTPases. *Annual Review of Pharmacology and Toxicology*.

[33] Bunney T. D., Harris R., Gandarillas N. L. (2006). Structural and mechanistic insights into ras association domains of phospholipase C epsilon. *Molecular Cell*.

[34] Pagel O., Loroch S., Sickmann A., Zahedi R. P. (2015). Current strategies and findings in clinically relevant post-translational modification-specific proteomics. *Expert Review of Proteomics*.

[35] Mocellin S., Verdi D., Pooley K. A., Nitti D. (2015). Genetic variation and gastric cancer risk: A field synopsis and meta-analysis. *Gut*.

[36] El-Serag H. B., Mason A. C., Petersen N., Key C. R. (2002). Epidemiological differences between adenocarcinoma of the oesophagus and adenocarcinoma of the gastric cardia in the USA. *Gut*.

[37] Powell J., McConkey C. C. (1992). The rising trend in oesophageal adenocarcinoma and gastric cardia. *European Journal of Cancer Prevention*.

[38] Wang L., Bi X., Song X. (2013). A sequence variant in the phospholipase C epsilon C2 domain is associated with esophageal carcinoma and esophagitis. *Molecular Carcinogenesis*.

[39] Sorli S. C., Bunney T. D., Sugden P. H., Paterson H. F., Katan M. (2005). Signaling properties and expression in normal and tumor tissues of two phospholipase C epsilon splice variants. *Oncogene*.

